# Supplementary material for: SARS-CoV-2 beta variant substitutions alter spike glycoprotein receptor binding domain structure and stability
Source: J Biol Chem. 2021 Oct 29;297(6):101371. doi: 10.1016/j.jbc.2021.101371 (PMC8553658; doi:10.1016/j.jbc.2021.101371)
Supplement: Figure S1 [file mmc1.pdf]

SARS-CoV-2 Beta Variant Substitutions Alter Structure and Stability of the Spike Glycoprotein Receptor-Binding Domain

Daniel L. Moss and Jay Rappaport

Figure S1. ff14SB specific changes in RBD residue contacts.

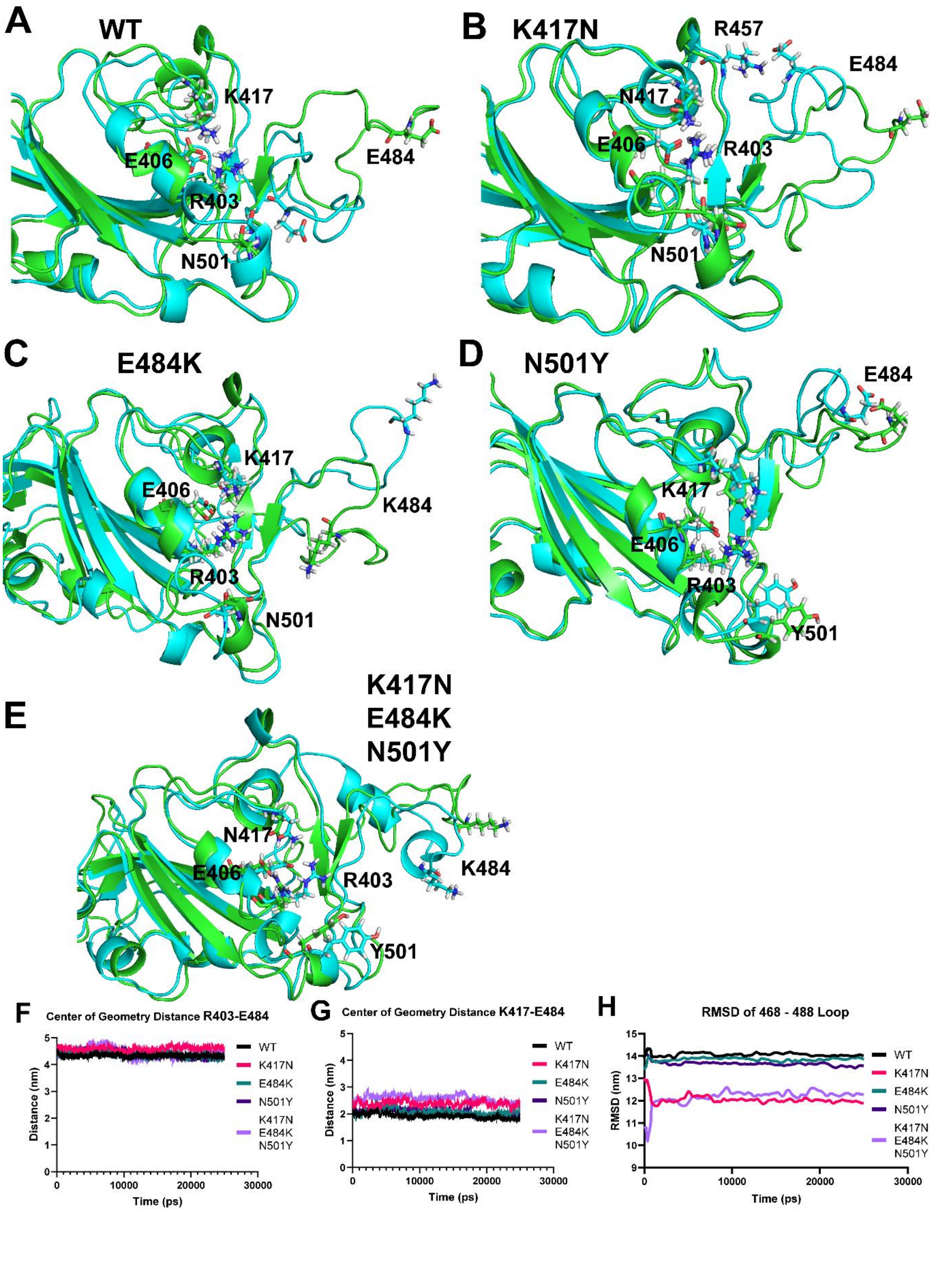
A
WT
K417
E406
R403
N501
E484
B
K417N
R457
E484
N417
E406
R403
N501
C
E484K
K417
E406
K484
R403
N501
D
N501Y
E484
K417
E406
R403
Y501
E
K417N
E484K
N501Y
N417
E406
R403
K484
Y501
F
Center of Geometry Distance R403-E484
Distance (nm)
Time (ps)
WT
K417N
E484K
N501Y
K417N E484K N501Y
G
Center of Geometry Distance K417-E484
Distance (nm)
Time (ps)
WT
K417N
E484K
N501Y
K417N E484K N501Y
H
RMSD of 468 - 488 Loop
RMSD (nm)
Time (ps)
WT
K417N
E484K
N501Y
K417N E484K N501Y

Figure S1. ff14SB specific changes in RBD residue contacts. A - E. RBD coordinates extracted from the molecular dynamics trajectories of the wild-type (A), K417N (B), E484K (C), N501Y (D) and K417N/E484K/N501Y (E) RBD variants in the ff14SB force field. The substituted residues as well as important hydrogen bonding pairs are shown. F. Center of geometry distance in nm between residue R403 and E484 plotted as a function of time for all RBD variants in the CHARMM36 force field. G. Center of geometry distance in nm between residue K417 and E484 plotted as a function of time for all RBD variants in the CHARMM36 force field. H. RMSD of the 468 – 488 loop relative to the starting structure for all RBD variants plotted as a function of time.
